# The Promoter of AtUSP Is Co-regulated by Phytohormones and Abiotic Stresses in *Arabidopsis thaliana*

**DOI:** 10.3389/fpls.2016.01957

**Published:** 2016-12-26

**Authors:** Monika Bhuria, Parul Goel, Sanjay Kumar, Anil K. Singh

**Affiliations:** ^1^Council of Scientific and Industrial Research – Institute of Himalayan Bioresource TechnologyPalampur, India; ^2^Academy of Scientific and Innovative ResearchNew Delhi, India

**Keywords:** abiotic stress, *Arabidopsis*, universal stress protein, *AtUSP*, phytohormones, promoter, deletion analysis

## Abstract

Universal stress proteins (USPs) are known to be expressed in response to various abiotic stresses in a wide variety of organisms, such as bacteria, archaebacteria, protists, algae, fungi, plants, and animals. However, in plants, biological function of most of the USPs still remains obscure. In the present study, *Arabidopsis* USP gene (*AtUSP*) showed induction in response to abscisic acid (ABA) and various abiotic stresses *viz*. heat, dehydration, salt, osmotic, and cold stresses. Additionally, *in silico* analysis of *AtUSP* promoter identified several *cis*-elements responsive to phytohormones and abiotic stresses such as ABRE, ERE, DRE, and HSE, etc. To functionally validate the *AtUSP* promoter, the 1115 bp region of promoter was characterized under phytohormone and abiotic stress treatments. Deletion analysis of promoter was carried out by cloning the full length promoter (D0) and its three 5′ deletion derivatives, D1 (964 bp), D2 (660 bp), and D3 (503 bp) upstream of the β-glucuronidase (GUS) reporter gene, which were then stably transformed in *Arabidopsis* plants. The *AtUSP* promoter (D0) showed minimal activity under non-stress conditions which was enhanced in response to phytohormone treatments (ABA and ACC) and abiotic stresses such as dehydration, heat, cold, salt, and osmotic stresses. The seedlings harboring D1 and D2 deletion fragments showed constitutive GUS expression even under control condition with increased activity almost under all the treatments. However, D3 seedlings exhibited complete loss of activity under control condition with induction under ACC treatment, dehydration, heat, oxidative, salt, and osmotic stresses. Thus, present study clearly showed that *AtUSP* promoter is highly inducible by phytohormones and multiple abiotic stresses and it can be exploited as stress inducible promoter to generate multi-stress tolerant crops with minimal effects on their other important traits.

## Introduction

During their life cycle, plants have to cope with various abiotic stresses, such as drought, salinity, high or low temperature, oxidative stress, etc. To counter these stresses, plants have evolved various stress tolerance and avoidance mechanisms. These mechanisms involve signal transduction cascades that respond to external stimuli by activating stress-responsive genes, which in turn bring about some morphological, physiological, and metabolic changes that help plants to survive these adverse conditions (Ahuja et al., 2010). In the field conditions, plants are often exposed to multiple stresses, simultaneously (Mittler, 2006), thus, there is a need to engineer plants with capability to survive and grow well under multiple abiotic stresses. In past, several studies have identified large subset of multiple abiotic stress-responsive genes, which might play important role in crosstalk between various stress signaling pathways (Kilian et al., 2007; Matsui et al., 2008).

The universal stress proteins (USPs) were first discovered in bacteria to be overexpressed in response to various stresses (Nystrom and Neidhardt, 1992). Accumulating evidences suggest that USPs are induced during multiple stresses and play important role in survival under abiotic stresses. For example, Nachin et al. (2005) showed the role of USP in survival of bacteria when exposed to various stress conditions, such as nutrient starvation and exposure to heat and oxidative stress. Furthermore, some studies showed induction of USPs in pathogenic microbes when exposed to immunological stresses of hosts, wherein they play important role in survival (Liu et al., 2007; Gomes et al., 2011). USPs are conserved among the archaebacteria, eubacteria, protozoa, fungi, plants, and metazoans (Kerk et al., 2003; Kvint et al., 2003; Isokpehi et al., 2011a). In *Escherichia coli*, six USP paralogs have been identified namely, UspA, UspC, UspD, UspE, UspF, and UspG (Kvint et al., 2003). Loss of function of UspA in bacteria was shown to have detrimental effect on bacterial growth during stationary phase (Nystrom and Neidhardt, 1993). Broadly, USPs have been classified into two groups; first group includes USPs with structure similar to UspA of *Haemophilus influenzae* with no ATP binding activity, while second group includes proteins with structural similarity to UspFG-type proteins of *Methanococcus jannaschii* that possess ATP binding activity. The USP proteins may be small, with single USP domain or may be large containing two tandem repeats of USP domains or may be present along with other functional domains such as Na^+^/H^+^ exchanger, amino acid permease, and protein kinase (Nachin et al., 2005). Kerk et al. (2003) identified 44 genes encoding USPA domain containing proteins in *Arabidopsis* and performed their phylogenetic analysis which revealed that they have evolved from 1MJH like ancestor. Since then, several USP proteins have been reported to play important roles in stress tolerance in various plants, such as *Arabidopsis*, rice, cotton, tomato, pigeonpea, and *Salicornia brachiata* (Sauter et al., 2002; Lenman et al., 2008; Merkouropoulos et al., 2008; Maqbool et al., 2009; Loukehaich et al., 2012; Udawat et al., 2014, 2016; Gonzali et al., 2015; Jung et al., 2015; Sinha et al., 2016). However, the precise function of most of the plant USPs has not been deciphered, so far.

From lower to higher organisms, the regulation of gene expression is the major factor that determines the adaptive capacity of an organism to various environmental stresses (Gasch et al., 2000; Balazi and Oltvai, 2005). In plants, spatial and temporal expression of specific genes is required to coordinate growth, development, and responses to various abiotic stresses. This tight regulation of gene expression occurs at the two levels, transcriptional and post-transcriptional. Multiple *cis*-acting regulatory elements present in the promoters of the genes are a type of transcriptional gene regulatory elements that are required for the specific expression pattern of a gene. Till date, various stress-responsive *cis*-elements have been identified in the promoters of stress inducible genes that allow their stress specific expression. The promoters of multiple stress-responsive genes have a number of regulatory elements that respond to multiple stresses, such as ABRE (abscisic acid-responsive element), LTRE (low temperature-responsive element), MYC and DRE (dehydration-responsive element) are the potential targets for regulating stress inducible expression of transgenes in transgenic plants (Maruyama et al., 2012). Plant responses to abiotic stresses, such as drought, salinity, cold, and heat are largely mediated by different hormonal signaling pathways that might act synergistically or antagonistically. Abscisic acid (ABA), ethylene and salicylic acid are the major phytohormones that act as link between plant responses to different abiotic stresses (Li et al., 2004; Fujita et al., 2006). Overexpression of stress-responsive genes under the control of CaMV35S promoter results in strong expression of genes that provide plants with stress tolerance. However, it might result in undesirable phenotypes, such as retarded and stunted growth and low seed yield (Liu et al., 1998). Several studies have shown that expression of stress-responsive genes driven under stress inducible promoter is far stronger than under CaMV35S promoter and that enhances plant stress tolerance with least effect on growth and yield of plant under normal conditions (Kasuga et al., 1999). Thus, in order to engineer multiple stress tolerant crop plants, it is necessary to identify and characterize multiple stress-responsive promoters, which may be used to regulate expression of transgenes. Since, many plants share similar transcriptional machineries and regulatory elements, therefore, *Arabidopsis* can be used as model system to characterize multiple stress-responsive promoters. Several of the bacterial UspA characterized till date provide resistance to multiple stresses (Nystrom and Neidhardt, 1992; Nachin et al., 2005; Liu et al., 2007). Till date, several plant USPs have been reported to be responsive to more than one stress. The *SpUSP* from wild tomato has been reported to be induced under ABA, ethylene, drought, salt, heat, wounding, oxidative, and cold stresses (Loukehaich et al., 2012). The expression of *SbUSP* gene from *Salicornia brachiata* has been shown to be induced by salt, drought, cold, and heat stress (Udawat et al., 2014). The promoter of cotton USP has been found to be responsive to dehydration, ABA, salt, heavy metals, gibberellic acid, and dark condition (Zahur et al., 2009). The ability of USPs to respond and provide tolerance against multiple stresses, suggests that their promoters might be good candidates to drive multiple stress-responsive expression of transgenes in transgenic plants.

In the present study, *AtUSP* expression was found to be induced under phytohormone and various abiotic stresses. Recently, overexpression of *AtUSP* has been shown to confer heat and oxidative stress tolerance in *Arabidopsis* (Jung et al., 2015). Therefore, the 1115 bp region upstream of the translation start site of *AtUSP* was cloned and functionally characterized in *Arabidopsis* through deletion analysis. Full length, *AtUSP* promoter showed least activity under non-stress conditions while its expression was highly induced by ABA, ACC, dehydration, heat, cold, salt, and osmotic stress. Full length *AtUSP* promoter showed tissue specific β-glucuronidase (GUS) expression which was lost in the subsequent deleted promoter fragments. This study showed that *AtUSP* promoter is multiple stress-responsive and can be used for guiding multiple stress-responsive expression of transgene in transgenic plants.

## Materials and Methods

### Plant Materials and Growth Conditions

Two week-old seedlings of wild type (WT; ecotype, Col-0) and transgenic *Arabidopsis thaliana* grown on ½ strength Murashige and Skoog (MS; Murashige and Skoog, 1962) medium supplemented with 1% sucrose and 0.8% agar at 22–23°C with 16/8h photoperiod and light intensity of 100 μmolm^-2^s^-1^ were used for *AtUSP* expression studies and promoter characterization under hormone and abiotic stress treatments, respectively.

### Stress Treatments

For expression and promoter analysis, 2 week-old WT and transgenic seedlings were subjected to different abiotic stress and hormone treatments. ABA (100 μM), ACC (ethylene precursor; 1-aminocyclopropane-1-carboxylic acid, 100 μM), salt (150 mM, NaCl), osmotic (300 mM, mannitol) and oxidative (50 μM methyl viologen) treatments were given by placing the seedlings on filter paper saturated with respective chemical in the ½ MS solution for 8 h (Tarte et al., 2015). Dehydration treatment was given by air drying seedlings on Whatman 3 MM paper for 30 min. Heat and cold treatments were given by incubating the seedlings on ½ MS medium at temperature of 37°C and 4°C for 8 h, respectively. Seedlings placed on filter paper saturated with ½ MS solution without any treatment were considered as control and each treatment was given in triplicate. After stress treatment, seedlings were harvested, frozen in liquid nitrogen and stored at –80°C till further use.

### RNA Isolation and Quantitative Real Time PCR Analysis

Total RNA was isolated from the frozen plant samples using iRIS method as described previously (Ghawana et al., 2011). First strand cDNA synthesis was carried out from 1 μg of total RNA using Verso cDNA synthesis kit (Thermo Scientific, USA). Real time primers for *AtUSP* (At3g53990) and GUS genes (**Supplementary File S1**) were designed using Primer Express 3.0.1 software (Applied Biosystems). The β-actin was used as internal control and the amplification specificity was determined by melt curve analysis. The expression analysis was performed with three biological and three technical replicates and relative gene expression was analyzed using 2^-ΔΔCT^ method as described previously (Livak and Schmittgen, 2001).

### Cloning of *AtUSP* Promoter and Its Deleted Fragments

A 1115 bp region of *AtUSP* promoter was amplified from genomic DNA of WT *Arabidopsis* and cloned into pGEM-T easy vector. Specific primers were designed with *Sal*I and *Nco*I sites in the forward and reverse primers, respectively, to amplify full length promoter (D0) excluding the coding sequence. Based on the position of different stress-responsive elements identified through *in silico* analysis, three 5′ promoter deletions were made by sequentially deleting 151, 455, and 612 bp from the 5′region and were named as D1, D2, and D3, respectively. Same reverse primer with *Nco*I site was used to amplify D0, D1, D2, and D3 fragments with *Sal*I site in the D0 and *Eco*RI site in the D1, D2, and D3 forward primers (**Supplementary File S2**). All these fragments were cloned into promoter-less vector pCAMBIA1391z fused with GUS gene and their schematic representation is depicted in **Supplementary Figure S1**.

### Sequence Analysis

The *AtUSP* promoter sequence was analyzed using PLACE^1^ and PLANTCARE^2^ databases to identify various *cis*-regulatory elements present in the *AtUSP* promoter.

### *Agrobacterium-*Mediated Transformation of *Arabidopsis* Plants

Four-week old *Arabidopsis thaliana* (WT) plants grown at 22–23°C with 16/8h photoperiod and light intensity of 100 μmolm^-2^s^-1^ were used for *Agrobacterium-*mediated transformation using vacuum infiltration method. For this, the unopened buds were dipped in the *Agrobacterium tumefaciens* (GV3101 strain) suspension in YEP medium with 30% sucrose and 0.2% Silvet-L77 solution by inverting the pots and applying the vacuum of 400 mm Hg for 5 min. The transformed plants were kept in the horizontal position in the dark for 24 h and then grown until the T_0_ generation seeds were harvested. T_0_ generation seeds were screened on 1X MS medium (1% sucrose and 0.8% agar) with hygromycin selection (20 μg/ml). The true transformants that were hygromycin resistant and able to reach the four leaf stage were transferred to pots containing a 1:1:1 mixture of vermiculite:perlite:coco peat. These plants were grown to maturity until the T_1_ generation seeds were harvested. Similarly, T_1_ and T_2_ seeds were germinated and plants were grown to maturity to harvest T_2_ and T_3_ generation seeds, respectively. Genomic DNA of eight T_3_ transgenic lines of *Arabidopsis* transformed with empty vector pCAMBIA1391z and recombinant vectors with D0, D1, D2, and D3 promoter fragments were isolated and analyzed for the presence of respective promoter fragment using PCR amplification (**Supplementary Figure S2**). The positive T_3_ transgenic *Arabidopsis* lines were used for functional characterization of *AtUSP* promoter and transgenic lines harboring empty vector pCAMBIA1391z were used as negative control.

### Histochemical Assay of GUS Activity

Histochemical GUS assay was carried out using the method as described previously (Jefferson, 1987). Two week old *Arabidopsis* seedlings with or without treatment were kept in GUS staining buffer containing 1 mM 5-bromo-4-chloro-3-indolyl β-D-glucuronidase (HiMedia), 100 mM sodium phosphate (pH 7.5), 0.5 mM potassium ferricyanide and 0.5 mM potassium ferrocyanide, 10 mM ETDA and 0.1 % (v/v) Triton-X 100 and a vacuum of 400 mm of Hg was applied for 5 min. Seedlings were incubated at 37°C overnight and cleared with 70% ethanol in order to remove chlorophyll for clear visualization of blue color of GUS stain. The stained seedlings were then observed and photographed under Carl-Zeiss Stereo DiscoveryV12 with AxioVision software.

### Quantitative Assay of GUS Activity

Quantitative assay was also performed as described by Jefferson (1987) using 4-methylumbelliferyl-β-D-glucuronide as substrate (MUG, Sigma). Protein was extracted from stress treated seedlings frozen in liquid nitrogen by grinding in GUS extraction buffer [50 mM sodium phosphate (pH 7.0), 10 mM β-mercaptoethanol, 10 mM EDTA, 0.1% (w/V) *N*-lauroyl sarcosine (SLS, Sigma), and 0.1% (v/v) Triton X-100). The tissue extract was centrifuged for 15 min at 4°C at 13000 rpm. The 10 μL of the protein extract was mixed with 390 μL of aliquoted GUS assay solution containing 22 mg MUG in 50 ml extraction buffer and incubated at 37°C for 1 h. The 100 μL of sample was taken from each tube at 0, 30, and 60 min incubation time, and 4.9 ml of 0.2 M Na_2_CO_3_ was added to stop the reaction. Fluorescence was measured with a fluorometer. Protein concentration was determined according to Bradford reagent using BSA standard (Bradford, 1976). Standard curve was prepared using 4-methylumbelliferone (4-MU, Sigma). GUS activity was expressed as nmol MU/min/mg protein.

### Statistical Analysis

The data is given in the form of mean of the values with standard deviation of replicates. Statistical analysis was carried out using Student’s *t*-test and *p*-values of <0.05 and <0.01 between treated and untreated samples were considered as significant and highly significant and marked with ^∗^ and ^∗∗^, respectively.

## Results

### Expression Analysis of *AtUSP* in Response to Hormone Treatment and Various Abiotic Stresses

**FIGURE 1:**
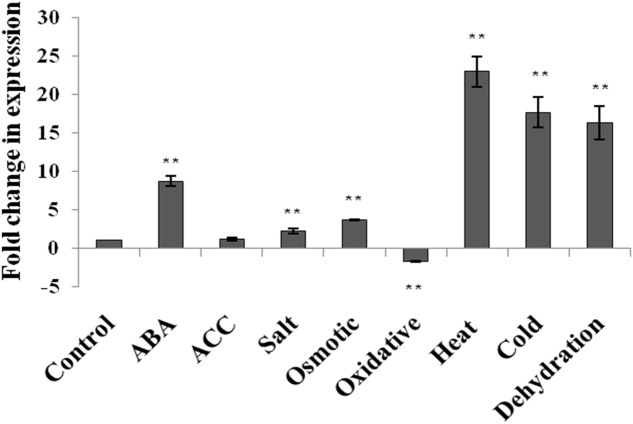
**Expression analysis of AtUSP in response to various phytohormones and abiotic stresses in 2 week old seedlings of *Arabidopsis thaliana*.** Data are represented as mean ± SD of replicates and statistical significance was determined by Student’s *t*-test (^∗^*P* < 0.05 and ^∗∗^*P* < 0.01).

The expression level of *AtUSP* gene was analyzed by qRT-PCR in 2-week old seedlings of *A. thaliana* (WT) in response to phytohormone treatments (ABA and ACC) and abiotic stresses such as dehydration, salt, osmotic, oxidative, heat, and cold stress (**Figure 1**). The expression of *AtUSP* was highly induced under ABA treatment and all the abiotic stress conditions, except for the oxidative stress. The highest induction was observed after heat exposure (22.9-fold) followed by cold (17.7-fold), and dehydration (16.4-fold). Salt and osmotic stresses also led to induction of *AtUSP* transcript levels by 2.3- and 3.7-fold, respectively (**Figure 1**; **Supplementary File S3**). ABA treatment resulted in 8.7-fold induction, while ACC treatment led to no significant change in the *AtUSP* expression. *AtUSP* gene expression was downregulated by 1.7-fold in response to oxidative stress.

### Cloning of *AtUSP* Promoter and Its Sequence Analysis

**FIGURE 2:**
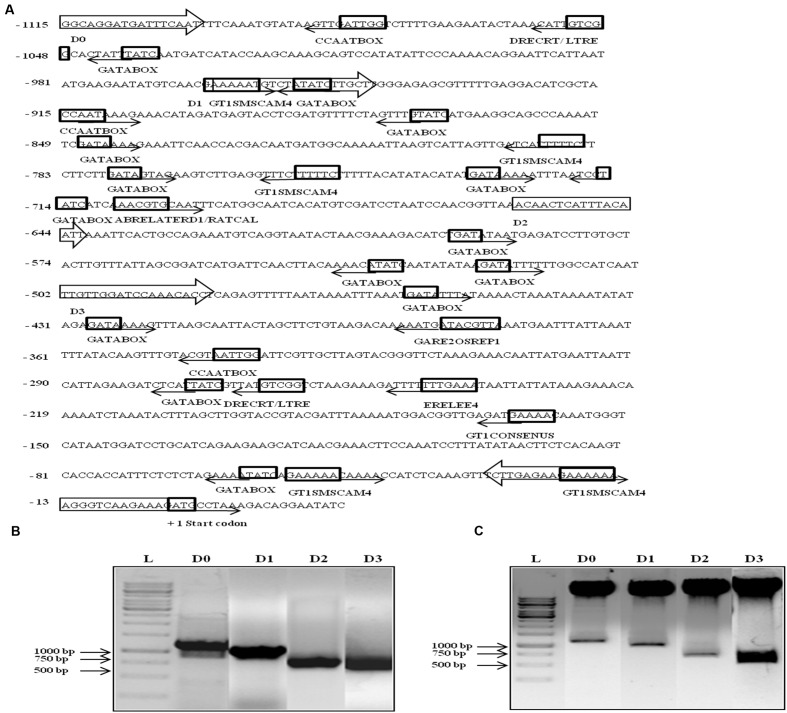
***In silico* analysis, amplification, and cloning of full length promoter of *AtUSP* (D0) and its 5′ deletion derivatives (D1, D2, and D3) into promoterless vector pCAMBIA1391z.** **(A)** Various putative *cis*-acting elements identified in full length (1115 bp) *AtUSP* promoter and its three 5′ deletion derivatives with arrows indicating the direction of forward and reverse primers. Arrows at the *cis*-acting elements indicate the position of elements on either + strand (forward direction) or - strand (reverse direction). +1 indicates the translation start site. **(B)** Amplification of full length (1115 bp) *AtUSP* promoter and its three deletion derivatives. **(C)** Restriction digestion of cloned D0, D1, D2, and D3 fragments in promoterless vector pCAMBIA1391z.

Since *AtUSP* was found to be multiple abiotic stress-responsive, its 1115 bp region upstream to translation start site was selected to characterize its promoter. This region was amplified from the genomic DNA of WT *Arabidopsis* and cloned in pGEM-T easy vector. The promoter sequence was analyzed using PLACE and PLANTCARE databases that led to identification of different putative *cis*-acting regulatory elements present in the *AtUSP* promoter (**Table 1**). The sequence motifs for ABA (ABRELATERD1 and ABRERATCAL), ethylene (ERELEE4), and gibberellin (GARE2OSREP1) were present at -700, -701, -238, and -377 positions, respectively (**Figure 2A**). Two motifs for salicylic acid (WBOXATNPR1) were present at positions -5 and -965. Five MYCCONSENSUSAT motifs at -153, -595, -678, -730, -1089, and one motif of each MYCATRD22 and MYBCONSENSUSAT were present at positions -678 and -662, respectively. Additionally, three motifs of MYBCORE elements known to be water stress-responsive were also present at -168, -659, and -662 positions. Five motifs of GT1GMSCAM4 responsible for pathogen and salt inducible expression were also found to be present in *AtUSP* promoter. It also contained two motifs of PREATPRODH known to be hypo-osmolarity responsive. *AtUSP* promoter had a number of *cis*-acting responsive elements for tissue specific expression such as CAATBOX1 and GATABOX present in several repeats (**Figure 2A**). It also contained certain motifs required for organ-specific expression, for example, PROXBBNNAPA for seed-specific expression, CACTFTPPCA1 for mesophyll cell-specific expression, RAV1AAT for high expression in rosette leaves and roots, RHERPATEXPA7 for root-hair specific expression, ROOTMOTIFTAPOX1 for root-specific expression, POLLEN1LELAT52 for pollen-specific expression, and TAAAGSTKST1 for guard cell-specific expression.

**Table 1:** Putative *cis*-acting elements present in the *AtUSP* promoter.

*cis*-element	Consensus	Number of repeats	Positions	Role	Reference
ACGTATERD1	ACGT	6	-342, -379, -702	Dehydration, dark induced senescence	Simpson et al., 2003
ARR1AT	NGATT	11	-102, -184, -212, -247, -333, -549, -667, -684, -715, -1077, -1103	Transcriptional activator ARR1 binding site	Ross et al., 2004
ABRELATERD1	ACGTG	1	-701	ABRE-like sequence	Nakashima et al., 2006
ABRERATCAL	MACGYGB	1	-700	ABRE related sequence	Kaplan et al., 2006
CAATBOX1	CAAT	14	-302, -337, -410, -502, -528, -642, -686, -697, -821, -910, -1033, -1051, -1076, -1098	Tissue specific expression of *LegA* gene in pea	Shirsat et al., 1989
CACTFTPPCA1	YACT	10	-205, -322, -406, -571, -610, -632, -771, -892, -1043, -1059	Mesophyll specific expression	Gowik et al., 2004
CBFHV	RYCGAC	3	-259, -675, -1046	Dehydration-responsive	Xue, 2002
DRECRTCOREAT	RCCGAC	2	-260, -1047	Drought, high salt, cold-responsive	Dubouzet et al., 2003
DRE2COREZMRAB17	ACCGAC	1	-259	ABA, Drought-responsive	Dubouzet et al., 2003; Kizis and Pages, 2002
EECCRCAH1	GANTTNC	2	-833, -987	Enhancer elements	Kucho et al., 2003
ERELEE4	AWTTCAAA	1	-237	Ethylene-responsive	Rawat et al., 2005
GATABOX	GATA	13	-54, -270, -382, -424, -455, -518, -531, -592, -711, -727, -773, -843, -867, -948, -1036	High level, light regulated, tissue specific expression	Reyes et al., 2004
GARE2OSREP1	TAACGTA	1	-377	Gibberellin responsive	Sutoh and Yamauchi, 2003
GT1CONSENSUS	GRWAAW	13	-14, -47, -57, -270, -422, -613, -725, -748, -785, -841, -958, -1036, -1094	Light-inducible, cell-specific expression	Zhou, 1999 Villain et al., 1996
GT1GMSCAM4	GAAAAA	5	-14, -47, -748, -785, -958	Pathogen and salt inducible	Park et al., 2004
CCAATBOX1	CCAAT	3	-336, -910, -1075	Heat stress-responsive	Rieping and Schoffl, 1992
LECPLEACS2	TAAAATAT	1	-433	Ethylene inducing xylanase (EIX) inducible	Matarasso et al., 2005
LTRECOREATCOR15	CCGAC	2	-261, -1047	Cold, drought, ABA-responsive	Baker et al., 1994
LTREATLTI78	ACCGACA	1	-259	Cold-responsive	Nordin et al., 1993
MYB1AT	WAACCA	1	-39	ABA, drought-responsive	Abe et al., 2003
MYB2CONSENSUSAT	YAACKG	1	-662	ABA, dehydration-responsive	Abe et al., 2003
MYBCORE	CNGTTR	3	-168, -659, -662	Water stress-responsive	Urao et al., 1993
MYCATERD1	CATGTG	1	-680	Dehydration-responsive	Simpson et al., 2003
MYCATRD22	CACATG	1	-678	ABA, Dehydration-responsive	Abe et al., 1997
MYCCONSENSUSAT	CANNTG	5	-153, -595, -678, -730, -1089	ABA, drought, cold-responsive	Abe et al., 2003; Chinnusamy et al., 2003
POLLEN1LELAT52	AGAAA	16	-2, -16, -26, -49, -59, -68, -221, -251, -306, 624, -747, -573, -784, -836, -877, -903	Pollen-specific expression	Bate and Twell, 1998
PREATPRODH	ACTCAT	2	-649, -892	Hypoosmolarity-responsive	Satoh et al., 2002
PROXBBNNAPA	CAAACACC	1	-484	Seed-specific, ABA-responsive expression	Ezcurra et al., 1999
RAV1AAT	CAACA	1	-496	High expression in rosette leaves, roots	Kagaya et al., 1999
RHERPATEXPA7	KCACGW	1	-699	Root-hair specific expression	Kim et al., 2006
ROOTMOTIFTAPOX1	ATATT	8	-144, -433, -453, -516, -526, -970, -979, -1003	Root-specific expression	Elmayan and Tepfer, 1995
TAAAGSTKST1	TAAAG	5	-98, -202, -224, -304, -906	Guard cell-specific expression	Plesch et al., 2001
WBOXATNPR1	TTGAC	2	-5, -965	Pathogen, salicylic acid inducible expression	Chen and Chen, 2002


### Development of Transgenic *Arabidopsis* Harboring *AtUSP* Promoter Constructs

The 1115 bp region just upstream of *AtUSP* translation start site was cloned in the promoterless vector pCAMBIA1391z at *Sal*I/*Nco*I sites and named as D0 construct containing the full length *AtUSP* promoter. Then, three deleted fragments were amplified by sequentially deletion of 151, 455, and 612 bp from the 5′ side of the full length promoter (**Figure 2B**). These fragments were then individually cloned into the promoterless vector pCAMBIA1391z at *Eco*RI/*Nco*I sites and were designated as D1 (964 bp), D2 (660 bp), and D3 (503 bp) constructs (**Figure 2C**). The D0, D1, D2, and D3 constructs were then transformed into *Arabidopsis* (WT) by *Agrobacterium*-mediated transformation using vacuum infiltration method. True transformants were first screened on 1X MS agar plates containing hygromycin and then further checked for the presence of desired fragment by PCR amplification using forward primer of each promoter fragment and reverse primer from GUS gene.

### Stress Induction of the GUS Gene Expression Driven by *AtUSP* Promoter at the Transcript Level

**FIGURE 3:**
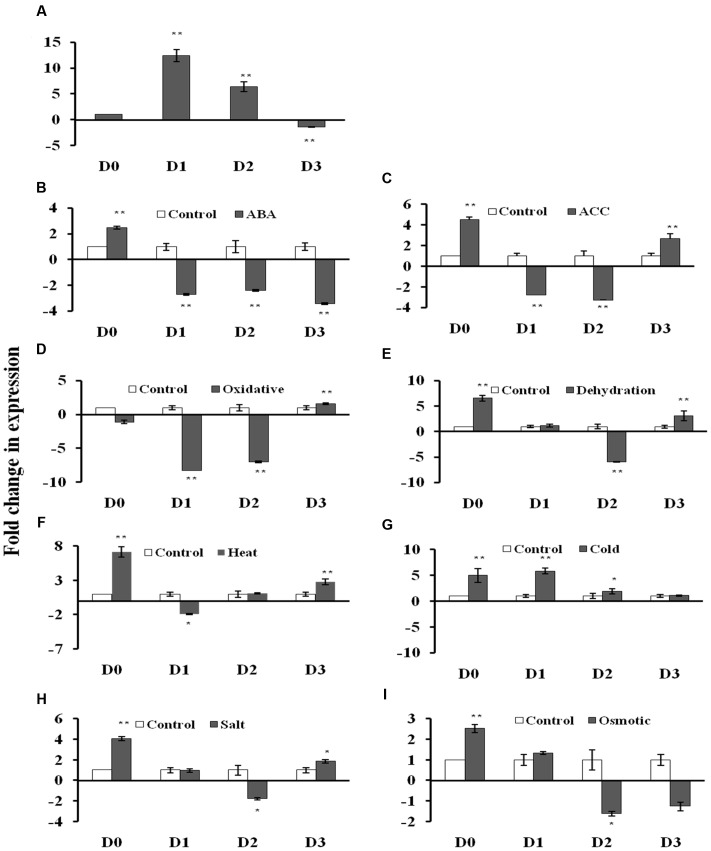
**β-glucuronidase (GUS) expression analysis in transgenic *A. thaliana* containing four different *AtUSP*:GUS constructs under control condition and various phytohormones and abiotic stress treatments.** **(A)** control condition, **(B)** abscisic acid (ABA) (100 μM), **(C)** ACC (100 μM), **(D)** oxidative stress (50 μM, methyl viologen), **(E)** Dehydration (air drying), **(F)** Heat (37°C), (**G**) Cold (4°C), **(H)** Salt (150 mM, NaCl), **(I)** Osmotic (300 mM, mannitol). Data are represented as mean ± SD of replicates and statistical significance was determined by Student’s *t*-test (^∗^*P* < 0.05 and ^∗∗^*P* < 0.01).

The GUS transcript level was measured using qRT-PCR in the 2 week old T_3_ seedlings of D0, D1, D2, and D3 transgenic *Arabidopsis* lines under non-stress and stress conditions (**Figures 3A–I**; **Supplementary File S4**). Under control conditions, the GUS gene showed 12.4- and 6.4-fold induction in the D1 and D2 lines, respectively, while 1.4-fold reduction in D3 lines with respect to D0 lines (**Figure 3A**). The D0 lines exhibited 2.5- and 4.5-fold GUS gene induction in response to ABA and ACC treatments, respectively. The D0 lines showed 7.2-, 6.6-, 5.0-, 4.0-, and 2.5-fold induction in the GUS gene expression under, dehydration, heat, cold, salt and osmotic stress, respectively (**Figures 3E–I**). While in case of oxidative stress, GUS expression was unaltered in D0 lines (**Figure 3D**). The D1 lines showed GUS gene induction only under cold (4.1-fold) stress with respect to D1 lines under non-stress conditions (**Figure 3G**). The GUS expression was downregulated in response to ABA, ACC, oxidative and heat stress in the D1 lines (**Figures 3B–D,F**). In case of D2 lines, the GUS expression was induced (1.9-fold) only under cold stress (**Figure 3G**), while, GUS expression was downregulated in response to ABA, ACC, oxidative dehydration, salt and osmotic stress (**Figures 3B–E,H,I**). The D3 seedlings showed induction in GUS expression in response to ACC treatment (2.7-fold), oxidative (1.6-fold), dehydration (3.1-fold), heat (2.8-fold), and salt stress (1.8-fold) with respect to D3 seedlings under non-stress conditions (**Figures 3C–F,H**). The GUS expression was downregulated by 3.4-fold in the D3 seedlings only under ABA treatment (**Figure 3B**).

### Histochemical GUS Assay for Phytohormone Treatment and Abiotic Stress-Responsiveness

**FIGURE 4:**
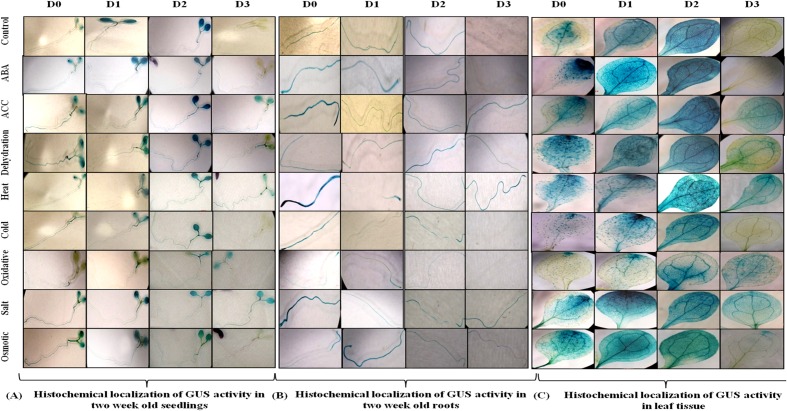
**Histochemical localization of GUS activity.** **(A)** In 2 week old seedlings **(B)** in root tissue and **(C)** in leaf tissue of transgenic *Arabidopsis* carrying full length *AtUSP* promoter (D0) and its three 5′ deletion derivatives (D1, D2, D3) in response to various phytohormones and abiotic stress treatments.

**FIGURE 5:**
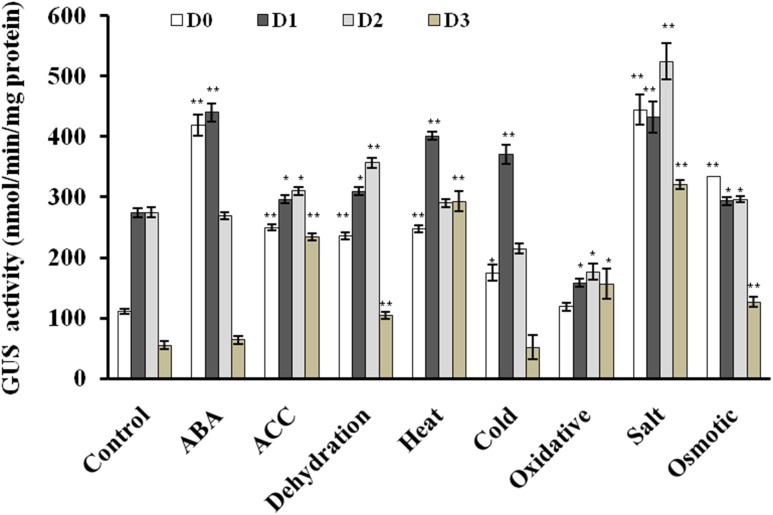
**Quantitative fluorometric assay for GUS activity in 2 week old seedlings in response to various phytohormones and abiotic stress treatments.** The experiment was carried out in three replicates. Data are represented as mean ± SD of replicates and statistical significance was determined by Student’s *t*-test (^∗^*P* < 0.05 and ^∗∗^*P* < 0.01).

Since *AtUSP* gene was found to be induced under multiple abiotic stresses, T_3_ generation of transgenic plants harboring *AtUSP* promoter and its three 5′ deleted fragments were analyzed for GUS induction under hormone treatments (such as ABA and ACC) and abiotic stresses (such as dehydration, salt, osmotic, heat, cold, and oxidative stress using histochemical staining and fluorimetric GUS assay (**Figures 4** and **5**). Under control conditions, strong GUS stain was detected in roots, while, a slight GUS staining was also detected in the region surrounding the mid-rib of the leaves, and hypocotyl of the D0 seedlings (**Figure 4**). In contrast, a strong GUS stain was observed in the entire leaf and root of the D1 and D2 seedlings under control condition. However, GUS stain was not detected in the D3 seedlings under control condition. Under ABA treatment, dark GUS stain was detected in the leaf tips, stomata, and roots of the D0 seedlings. However, in the D1 seedlings, GUS expression was found to be in the entire leaf, hypocotyls, and roots under ABA treatment. The GUS staining in D2 seedlings in response to ABA treatment was comparable to GUS stain found in untreated D2 seedlings which was evident by comparable GUS activities observed in the D2 seedlings under both the conditions (**Figures 4** and **5**). However, GUS staining was completely absent in D3 seedlings. ACC treatment resulted in slight GUS staining in the region surrounding mid-rib of leaves, hypocotyls, and roots in the D0 seedlings. Similar GUS staining pattern was observed in the leaves and roots of the D1 and D2 seedlings. ACC treated D3 seedlings also exhibited GUS staining in the leaves and roots. Dehydration stress also resulted in similar GUS staining pattern as that observed in case of ABA treatment. Strong GUS expression was observed in the leaf tip and stomata as in case of ABA treated D0 lines. The D1 and D2 seedlings also showed GUS expression following dehydration. However, in contrast to ABA treatment, D3 seedlings also exhibited GUS staining in response to dehydration, which was further confirmed by GUS activity assay with 1.9-fold increase in GUS activity observed under dehydration (**Figure 5**; **Supplementary File S5**). In D0 and D1 seedlings, GUS stain was detected in the region surrounding the leaf tips, hypocotyl, stomata, and roots under heat stress. Similar GUS staining was detected in D1 seedlings except for roots as GUS stain was detected only in root apex. Comparatively, weaker expression was observed in D2 and D3 seedling under heat stress, where GUS stain was detected in the entire leaf, hypocotyls, and roots. With cold treatment, GUS stain was detected only at the leaf tip, hypocotyl, stomata, and roots in the D0 seedlings. The tissue specific cold induction in GUS expression was observed in D1 seedlings with stronger expression in the region surrounding the leaf tip, stomata, hypocotyl with weak GUS expression in the roots. There was complete loss of GUS staining in the D3 seedlings under cold stress. Oxidative stress resulted in the weak GUS expression at the leaf tips, stomata, and roots of D0 and D1 seedlings as compared to control conditions. Interestingly, significant GUS activity (2.8-fold) was detected in D3 seedlings when subjected to oxidative stress (**Figure 5**). Under salt stress, GUS stain can be detected in the region surrounding the leaf tip, mid-rib region, hypocotyl, and in the entire root of the D0 seedlings. Similar GUS stain was also detected in the D1 seedlings with lower staining in the roots. Strong GUS staining was also observed in the salt treated D2 and D3 seedlings. Osmotic stress also resulted in similar GUS expression pattern in D0 seedlings as observed under salt stress, except in roots where GUS staining was found only in the root tip. Slight induction in GUS expression was observed in the D1 and D2 seedlings, while, weaker induction was still observed in D3 seedlings (**Figure 4**).

## Discussion

Universal stress proteins, well known for their universal expression under different stresses in numerous plants are considered as potential targets for developing stress tolerant plants. Expression of several plant *USP* genes has been shown to be induced under various abiotic stresses, but their precise molecular function is still unknown (Sauter et al., 2002; Maqbool et al., 2009; Loukehaich et al., 2012; Gonzali et al., 2015; Jung et al., 2015; Sinha et al., 2016; Udawat et al., 2016). Two plant USPs, i.e., *SpUSP* from wild tomato and *SbUSP* from *S. brachiata* have been reported to be induced in response to multiple abiotic stresses (Loukehaich et al., 2012; Udawat et al., 2014). In addition, cotton USP promoter also showed enhanced activity under ABA, dehydration, and salt treatments (Zahur et al., 2009). These studies suggest that promoters of USP genes have the potential to be used as stress inducible promoter for the development of multiple stress tolerant crops. In present study, *AtUSP* gene was found to be induced under phytohormone and abiotic stress treatments. *AtUSP* gene was highly induced by abiotic stresses such as dehydration, cold, heat, salt, osmotic stress and phytohormone, ABA. Our results are in accordance with a previous microarray study which reported that *AtUSP* was upregulated under drought stress (Isokpehi et al., 2011b). In another report, AtUSP protein level was enhanced in response to cold acclimation that was responsible for enhanced freezing tolerance (Kawamura and Uemura, 2003). Recently, *AtUSP* was shown to have chaperone like function which is regulated by ROS level and provide resistance to heat shock and oxidative stress (Jung et al., 2015).

Based on the multiple stress-responsive characteristic of *AtUSP*, full length promoter (1115 bp) and its three 5′ deletion derivatives were generated and used to drive expression of GUS reporter gene by cloning them in a promoterless vector pCAMBIA1391z. These constructs were then transformed into WT *A. thaliana* by *Agrobacterium tumefaciens-*mediated transformation. The 1115 bp region of *AtUSP* promoter contained several *cis-*regulatory elements required for tissue specific, hormone, and stress-responsive expression. *AtUSP* promoter contained MYCCONSENSUSAT, MYCATRD22, MYBCORE, and MYBCONSENSUSAT elements which were reported to be also present in the promoter of dehydration-responsive RD22 that is known to play role in ABA, drought and cold signaling (Abe et al., 1997, 2003). Several repeats of CAATBOX1 and GATABOX motifs required for tissue specific expression were also present (Shirsat et al., 1989). ABA-responsive elements, such as ABRELATERD1, ABRERATCAL, ethylene-responsive element (ERELEE4), and gibberellic acid-responsive element (GARE2OSREP1) were also present in the *AtUSP* promoter, which are responsible for ABA, ethylene and gibberellic acid induced expression, respectively (Sutoh and Yamauchi, 2003; Rawat et al., 2005; Kaplan et al., 2006; Nakashima et al., 2006). GT1GMSCAM4 motifs known for pathogen and salt induced expression (Park et al., 2004) were also present. The *AtUSP* promoter analysis was in agreement to earlier report, which suggested that the ∼0.5 kb region of promoter of multiple stress-responsive genes tend to contain large number of *cis-*regulatory elements responsive to multiple stresses with several unique elements (Walther et al., 2007). For D0 and D3 seedlings, the GUS transcript level strongly correlated with the respective GUS activity. For D1 and D2 seedlings, there was no strong correlation between the GUS transcript and the corresponding GUS activity under different phytohormone and stress treatments. This might be due to the fact that we compared GUS expression in the D1 and D2 seedlings under various stresses with respect to their respective controls and moreover, the basal expression levels of GUS gene in the D1 and D2 seedlings were already high under control conditions. GUS staining significantly correlated with the measured GUS activity in the non-stressed and stressed transgenic seedlings. GUS histochemical staining as well as GUS activity assay clearly revealed that D0 seedlings possess minimal activity under non-stressed condition and oxidative stress. However, the D1 and D2 seedlings showed much stronger GUS expression with respect to D0 seedlings under non-stressed condition. The intense spread of GUS stain in the entire leaf tissue of the D1 and D2 seedlings can be due to the deletion of four motifs of CAAT box and one motif of GATABOX that have been shown to be required for tissue specific expression (Shirsat et al., 1989; Reyes et al., 2004). The complete loss of GUS expression in the D3 seedlings under control conditions might be due to deletion of 10 motifs of GATABOX required for high level transcription, RAV1AAT responsible for high expression in leaves and five motifs of ROOTMOTIFTAPOX1 required for root-specific expression (Elmayan and Tepfer, 1995; Kagaya et al., 1999; Reyes et al., 2004). Under control conditions, strong GUS expression in the roots of D0 seedlings might be due to the presence of eight ROOTMOTIFTAPOX1 motifs which is responsible for strong expression in roots (Elmayan and Tepfer, 1995). The full length *AtUSP* (D0) promoter was strongly activated by ABA, ACC, dehydration, salt, osmotic, heat, and cold stresses. These results strengthen our qRT-PCR based gene expression analysis which also highlighted the multiple stress-responsive characteristic of *AtUSP*. Recently, Jung et al. (2015), showed that *AtUSP* possess molecular chaperone like function which is regulated by ROS homeostasis. They also showed that AtUSP plays an essential role in heat and oxidative stress tolerance in *Arabidopsis.* ABA accumulation has been shown to act as trigger to mediate stress tolerance during dehydration, salt, and cold stresses (Finkelstein and Lynch, 2000). Therefore, this might provide the possible explanation for similar GUS expression pattern observed in response to ABA and dehydration. Plant responses include both ABA-dependent and ABA-independent mechanisms that help to overcome abiotic stress conditions. ABA-dependent pathway includes many ABA-inducible genes that contain ABRE elements in their promoters (Mundy et al., 1990). *AtUSP* promoter also contains two motifs of ABRE-related sequences that might be responsible for ABA-mediated stress induction of GUS activity in D0 and D1 seedlings. Although, higher GUS activity was observed in the D1 seedlings in response to ABA, however, at the transcript level, GUS expression was downregulated in the D1 seedlings under ABA treatment, which might be attributed to higher GUS expression observed in the D1 seedlings under non-stress conditions (**Figure 3A**). ABA induction of GUS activity was lost in the D2 and D3 seedlings with deletion of two ABRE motifs. ABA-independent signaling involves dehydration-responsive elements in promoter region that are bound by DRE binding transcription factors (DREBs/CBFs) to induce the expression of downstream genes. Several studies have shown that overexpression of DREB/CBF lead to drought, salinity, and freezing tolerance in crops (Liu et al., 1998; Datta et al., 2012; Pierre et al., 2012). *AtUSP* promoter also contains several DRE elements that might be responsible for its multiple stress-responsive characteristic. D3 seedlings also showed weak induction with respect to non-stress conditions in response to dehydration which might be due to the fact that it still contains dehydration-responsive elements such as MYCCONSENSUSAT, LTRECOREATCOR15, DRECRTCOREAT, CBFHV, and DRE2COREZMRAB17 which have been shown to be part of ABA-independent signaling. Strong induction of GUS expression in D0 and D1 seedlings under osmotic stress in comparison to their respective controls can be attributed to presence of all the motifs of ABRE, MYB2CONSENSUSAT, MYCCONSENSUSAT, DRECRTCOREAT, and DRE2COREZMRAB17 which are directly or indirectly involved in osmotic stress response. Ethylene has been shown to play a positive role in mediating the plant salt tolerance (Cao et al., 2006). Similar GUS expression pattern was observed in the ACC and salt treated transgenic seedlings. Weak GUS induction observed in the D3 seedlings upon salt treatment even with the deletion of GT1GMSCAM4 might be due to the presence of ERE (ERELEE4) element. Similar weak induction can be seen in case of ACC treatment of D3 seedlings which reinforced the fact that ethylene plays a positive role in plant salt tolerance (Cao et al., 2006). Stronger induction in GUS activity was observed in the D0 and D1 seedlings while weak induction was observed in case of D2 seedlings under heat stress as compared to their respective controls. Heat stress induction of GUS activity was also observed in the D3 seedlings as this construct still retained one of the three heat stress-responsive elements responsible for heat induced expression (Rieping and Schoffl, 1992). Strong cold induction in GUS expression was observed only in D0 and D1 seedlings. The deletion of three motifs of MYCCONSENSUSAT in the D2 and D3 constructs might have resulted in the loss of cold induction in GUS expression in the D2 and D3 seedlings. Comparatively, lower GUS stain was detected in the D0, D1, and D2 seedlings under oxidative stress as compared to their respective controls. However, GUS expression was found to be increased in case of D3 seedlings as compared to non-stress condition. This might be due to the deletion of a negative regulatory region present in the promoter sequence in between -1115 and -504 bp region in the D3 seedlings for oxidative stress. This negative regulatory region might be responsible for the observed downregulation of *AtUSP* gene under oxidative stress.

## Conclusion

The present study reveals the multiple stress-responsive characteristic of *AUSP* gene and its promoter was functionally characterized under phytohormone and abiotic stress treatments. The GUS expression and histochemical studies showed that *AtUSP* promoter can respond to phytohormones such as ABA, ethylene, and several abiotic stresses such as dehydration, salt, osmotic, heat, and cold stress. Therefore, the D0 promoter fragment can be considered as potential multiple stress inducible promoter to derive the expression of transgenes that may confer stress tolerance to the plants.

## Author Contributions

AS conceived and designed the experiments. MB and PG performed the experiments. MB and AS performed the data analysis. AS and SK secured the funds to support this research. MB wrote the manuscript. MB and AS revised and finalized the manuscript.

## Conflict of Interest Statement

The authors declare that the research was conducted in the absence of any commercial or financial relationships that could be construed as a potential conflict of interest.

## Footnotes

1 http://www.dna.affrc.go.jp/PLACE/

2 http://bioinformatics.psb.ugent.be/webtools/plantcare/html/
