# Supplementary material for: The Promoter of AtUSP Is Co-regulated by Phytohormones and Abiotic Stresses in Arabidopsis thaliana
Source: Front Plant Sci. 2016 Dec 26;7:1957. doi: 10.3389/fpls.2016.01957 (PMC5183650; doi:10.3389/fpls.2016.01957)
Supplement: Supplementary file 6 [file Image_1.PDF]

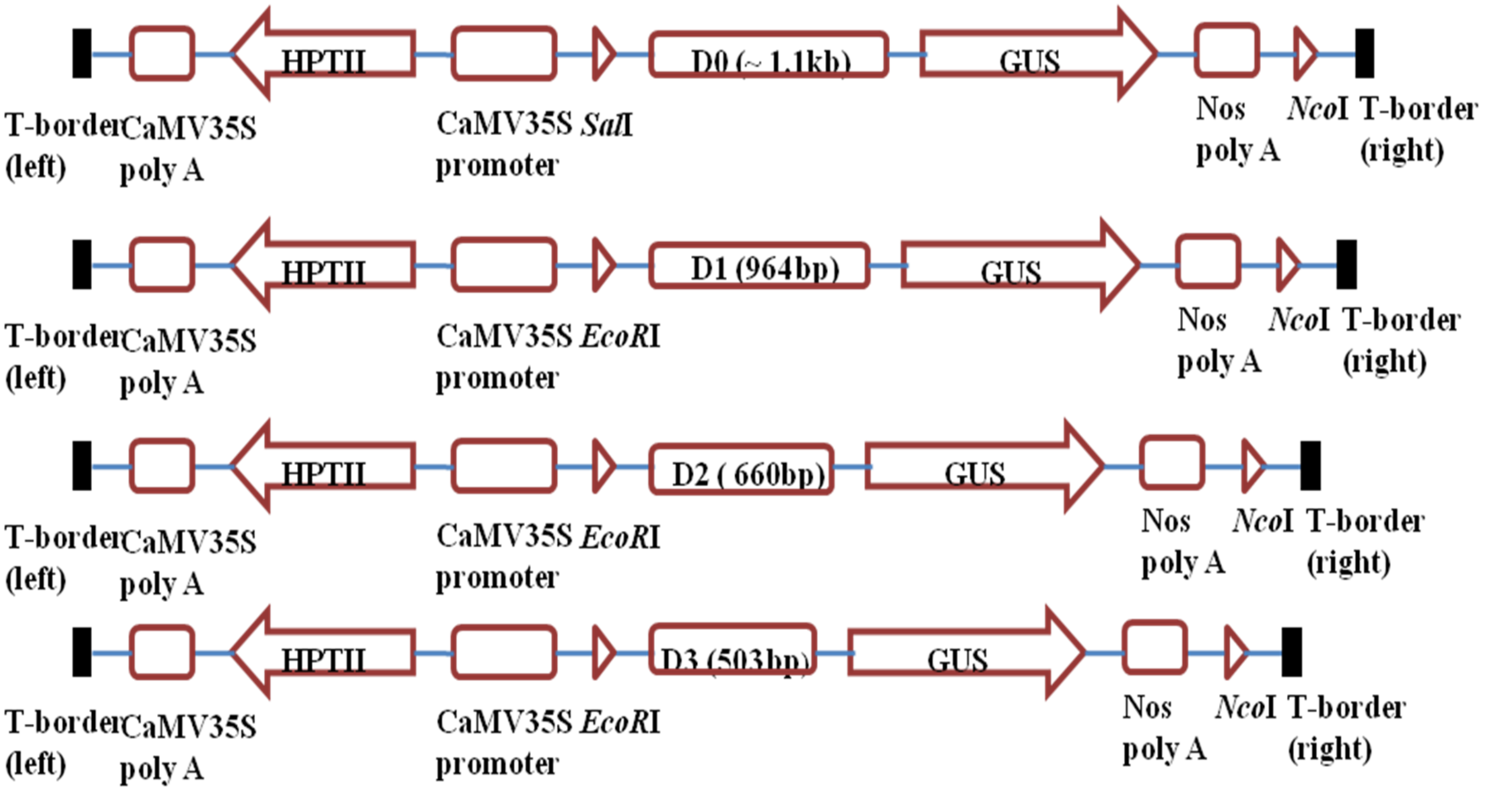


**Supplementary Figure S1:** Schematic representation of full length promoter (D0) and its three 5′deletion derivatives (D1, D2, D3) cloned into pCAMBIA1391z vector at *Sal*I and *Nco*I or *Eco*RI and *Nco*I restriction sites, respectively.
