# Supplementary material for: The Promoter of AtUSP Is Co-regulated by Phytohormones and Abiotic Stresses in Arabidopsis thaliana
Source: Front Plant Sci. 2016 Dec 26;7:1957. doi: 10.3389/fpls.2016.01957 (PMC5183650; doi:10.3389/fpls.2016.01957)
Supplement: Supplementary file 7 [file Image_2.PDF]

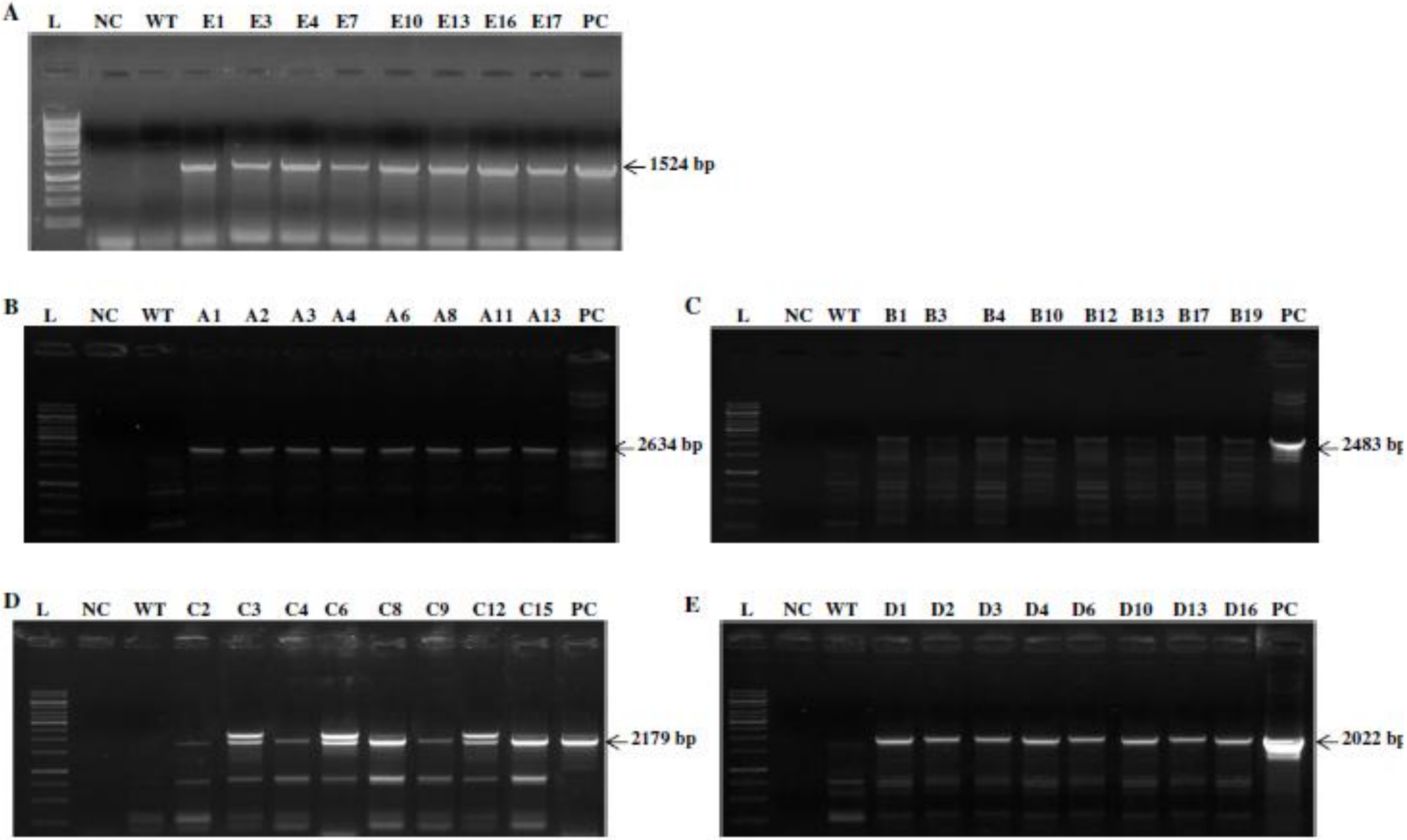

Supplementary Figure S2: PCR confirmation of putative transgenic *Arabidopsis* lines harboring **(A)** empty pCAMBIA1391z vector **(B)** D0 **(C)** D1 **(D)** D2 **(E)** D3 fragments cloned in pCAMBIA131z vector using genomic DNA as template with forward primer of respective promoter fragment and GUS gene reverse primer; L: 1 kb ladder; NC: negative control; WT: wild -type *Arabidopsis*; E1to E17: transgenic lines of empty vector pCAMBIA1391z; A1 to A13: transgenic lines of D0-pCAMBIA139z construct; B1 to B19: transgenic lines of D1-pCAMBIA139z construct; C2 to C15: transgenic lines of D2-pCAMBIA139z construct and D1 to D16: transgenic lines of D3-pCAMBIA139z construct.
